# Prevalence and pathology of equine parvovirus-hepatitis in racehorses from New York racetracks

**DOI:** 10.1186/s12985-022-01901-3

**Published:** 2022-11-01

**Authors:** Mason C. Jager, Joy E. Tomlinson, Caitlin E. Henry, Megan J. Fahey, Gerlinde R. Van de Walle

**Affiliations:** Baker Institute for Animal Health, College of Veterinary Medicine, Cornell University, 14853 Ithaca, NY USA

**Keywords:** Equine parvovirus-hepatitis, Racehorses, Liver samples, *In situ* hybridization, Prevalence, Hepatitis

## Abstract

**Background:**

Theiler’s disease, a.k.a. equine serum hepatitis, is a devastating, highly fatal disease of horses. Equine parvovirus-hepatitis (EqPV-H) has been identified as the likely cause of this disease. While the incidence of Theiler’s disease is low, the prevalence of EqPV-H DNA in horses is high, with up to 37% in some regions, suggesting that subclinical or persistent infection is common.

**Methods:**

To determine the prevalence and pathogenicity of EqPV-H infection at New York racetracks, DNA was extracted from archived formalin-fixed, paraffin-embedded liver tissues from racehorses submitted for necropsy to the Animal Health Diagnostic Center as part of the New York State Gaming Commission-Cornell University postmortem examination program. A total of 191 liver samples from horses between 2 and 13 years old were evaluated. Extracted DNA was tested for EqPV-H using PCR and gel electrophoresis. PCR-positive samples were further assessed for tissue morphology using histology and detection of viral nucleic acid using in situ hybridization.

**Results:**

Forty-two samples were PCR positive (22%). Of those, 31 samples had positive viral nucleic acid hybridization in hepatocytes with 11 samples showing positive hybridization in necrotic hepatocytes associated with inflammatory cells, indicating active hepatitis. Both individual hepatocyte necrosis and hepatitis were positively associated with EqPV-H detection (p < 0.0001 and p = 0.0005, respectively).

**Conclusion:**

These findings indicate that presence of EqPV-H in the liver and parvoviral-associated hepatitis are prevalent in racehorses from New York racetracks, thus warranting additional studies examining potential associations between EqPV-H infection and racehorse performance.

**Supplementary Information:**

The online version contains supplementary material available at 10.1186/s12985-022-01901-3.

## Background

Equine parvovirus-hepatitis (EqPV-H) was first reported in 2018 in liver and serum samples of a horse that died of Theiler’s disease following administration of tetanus antitoxin and has been identified as the likely cause of this disease through case series and experimental infections [1–16]. EqPV-H has a small, single-stranded DNA genome of approximately 5.3 kb and has been assigned to the species *Ungulate Copiparvovirus 6* in the genus *Copiparvovirus* based on genome organization and genetic relatedness to other parvoviruses [1, 17]. Two large open reading frames are predicted to encode a nonstructural protein (NS) and a capsid protein (VP), although a detailed transcriptome profile has yet to be done for EqPV-H [1]. To date, EqPV-H is the only member of *Copiparvovirus* known to cause clinical disease [18].

Experimental EqPV-H infections of horses demonstrated hepatotropism and mild or subclinical hepatitis, but no recapitulation of naturally occurring Theiler’s disease [1, 2]. Studies analyzing serum samples from clinically healthy horses across the world have shown an EqPV-H DNA prevalence between 7.1% and 37% and a seroprevalence between 15% and 34.7% [1–16]. Collectively, these studies suggest that EqPV-H is endemic among horse populations and that the most common manifestation of EqPV-H infection is subclinical to mild hepatitis.

In racehorses specifically, a low prevalence of EqPV-H in clinically healthy animals has been reported with an association with sex and decreased performance, but not with serum biochemistry abnormalities, in a recent study in South Korea [14]. Another study of 60 apparently healthy racehorses in China detected EqPV-H DNA in 8.33% of these horses, with mild elevations in serum liver enzymes in two of the EqPV-H-positive animals [6]. Lastly, a study of elevated gamma-glutamyl transferase (GGT) syndrome in Thoroughbred racehorses from New York, Florida, and Kentucky, found that 37% of serum samples from case and control horses were positive for EqPV-H DNA by PCR [10]. Despite this widespread detection of EqPV-H in racehorses, very little is known whether EqPV-H infection is associated with histologically confirmed hepatitis. Thus, the goal of this present study was two-fold: [1] to assess the prevalence of EqPV-H in racehorses from New York racetracks using liver samples and [2] to determine whether infection was associated with liver pathology.

## Materials and methods

### Case selection

A total of 191 liver samples were collected from racehorses that experienced racing, training, or non-exercise fatalities at 10 New York Racing Association (NYRA) racetracks between January 1, 2016 and December 31, 2019 and were submitted for necropsy to the Animal Health Diagnostic Center (AHDC) as part of the New York State Gaming Commission (NYSGC)-Cornell University postmortem examination program. Collection of formalin-fixed, paraffin-embedded (FFPE) liver tissue, in addition to heart, skeletal muscle, spleen, lung, or kidney tissue, was part of the routine for each necropsy and was collected within 24 h of euthanasia or death. Only horses with a minimum of 0.5 cm^2^ FFPE liver tissue were included in this study.

### Molecular characterization

DNA was purified from three 10 µm-thick scrolls from all 191 FFPE liver tissues using the QIAamp DNA FFPE Tissue Kit (Catalog no. 56404 Qiagen, Hilden, Germany), according to manufacturer’s instructions. PCR of the putative VP region of the genome was performed on DNA extracts using the forward primer sequence 5’- CACGGTCCCAGGACATTTAC and the reverse primer sequence 5’- TCACAGATCGTCCCTACCAC, as previously described, with an expected amplicon size of 87 base pairs (bp) [3, 4]. Gel electrophoresis was performed on amplified DNA and amplicon size was compared to both positive and negative controls. Positive control tissue consisted of FFPE-embedded liver from an experimentally infected horse, confirmed by both qPCR and in situ hybridization (ISH) to be EqPV-H positive, and negative control tissue consisted of FFPE-embedded liver from an EqPV-H qPCR-negative horse that was collected prior to its enrollment in an experimental infection study [2].

### Histopathology

Histopathology was performed on all EqPV-H PCR-positive liver tissues and on approximately twice as many PCR-negative liver tissue controls that were matched for age, sex, and breed. Tissue Sect. (4 μm) of FFPE-embedded liver were analyzed after staining with hematoxylin and eosin (H&E) by a board-certified anatomic pathologist (MJ), blinded to PCR status. Tissues were assessed for the presence of shrunken, hypereosinophilic individual necrotic hepatocytes associated with inflammatory infiltrates, and hepatitis was defined as any case with greater than two lobular infiltrates of inflammatory cells that included more than four lymphocytes, macrophages, or neutrophils. Cases with severe autolysis artifacts were not assessed for hepatitis or individual necrotic cells.

### In situ hybridization (ISH)

ISH was performed on all EqPV-H PCR-positive liver tissues, using the RNAScope® probe V-EqPV-H-VP1 (Catalog no. 559,991, Advanced Cell Diagnostics, Inc., Newark, CA, USA) against the EqPV-H *VP1* gene, exactly as previously described [2, 12]. Scoring was assigned based on following criteria: (+) = 1–2 positive cells per tissue section, (++) = 3–10 positive cells per tissue section, and (+++) = greater than 10 positive cells per tissue section with at least 1–3 positive cells per hepatic lobule.

### Statistical analysis

Logistic regression was used to assess whether age, sex, breed, season, or musculoskeletal injury were associated with increased risk of liver being EqPV-H PCR positive, and whether liver being EqPV-H PCR positive was associated with increased risk of individual hepatocyte necrosis and/or hepatitis. The association between liver being EqPV-H PCR positive and facility was assessed separately with a Chi-square analysis due to complete segregation of facilities by breed. Analyses were performed in JMP® Pro Version 14.0. Significance was set at p < 0.05.

## Results

### Equine parvovirus-hepatitis (EqPV-H) is prevalent in racehorses from New York racetracks

**Fig. 1:**
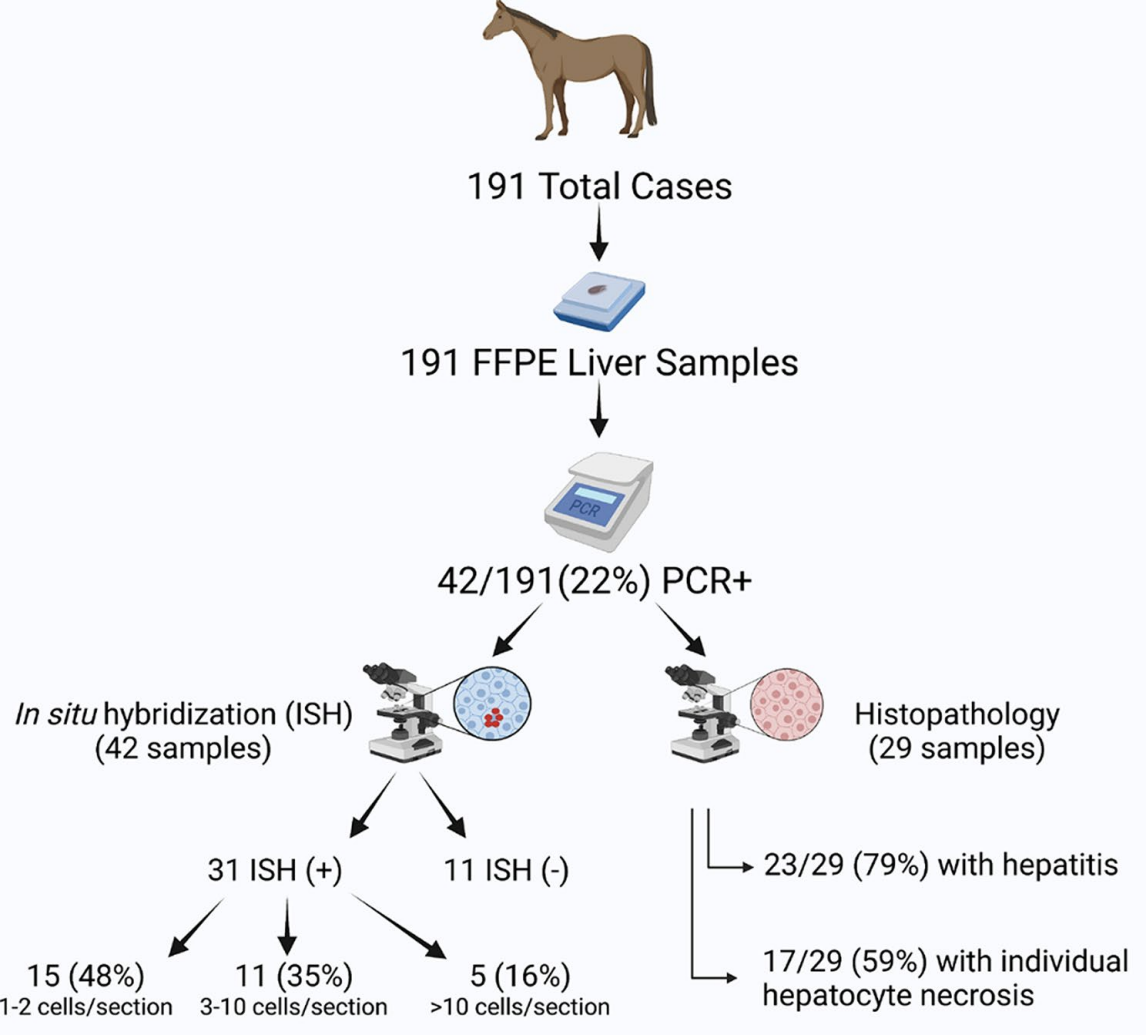
**Schematic overview of study design.** All 191 cases examined in this study were evaluated for EqPV-H infection using PCR of formalin-fixed paraffin-embedded (FFPE) scrolls. PCR-positive cases (42) were further analyzed by in situ hybridization (ISH) to evaluate the distribution and frequency of infected cells. Of these PCR-positive cases, 31 had evidence of hybridization. They were also analyzed by histology, with 29 samples of sufficient quality for histopathological analysis

In this retrospective study, liver tissue samples were collected during routine necropsy of 191 racehorses between 2016 and 2019 (Fig. 1). The horse sample population consisted of 157 Thoroughbreds and 34 Standardbreds and included 32 stallions, 87 geldings, and 72 mares. The median age was 4 with a range of 2–13 (Table 1).

**Fig. 2:**
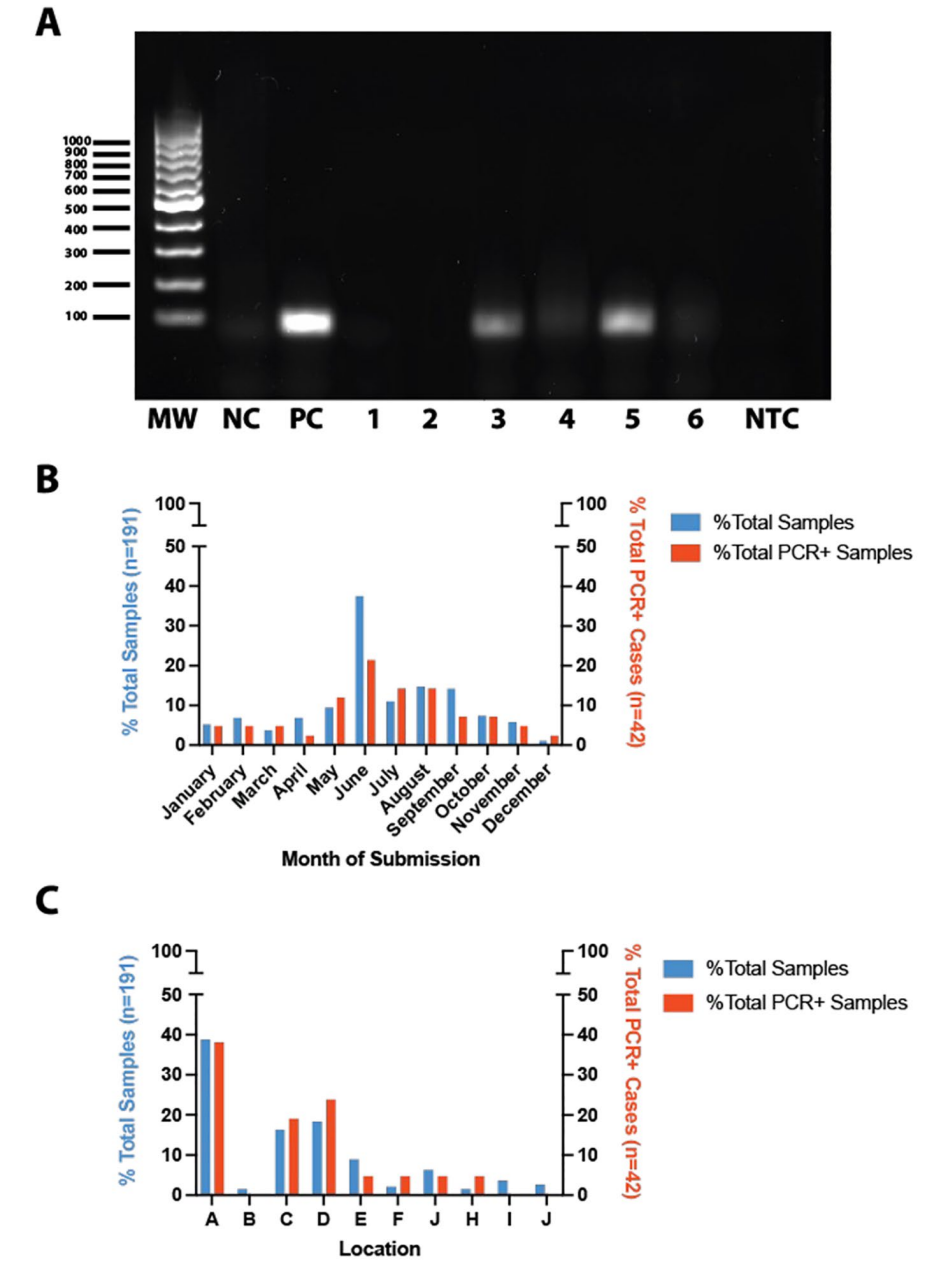
**Representative gel electrophoresis and distribution of cases by location and month of submission. (A).** A representative electrophoresis gel following PCR of DNA extracted from FFPE. MW = MW ladder, NC = negative control, PC = positive control, NTC = no template control, lanes 1–6 are samples. Lanes three (Case 27) and five (Case 34) were interpreted as positive with bands in the range expected for an 87 bp amplicon. **(B).** The percentage of total and PCR-positive samples submitted each month. **(C).** The percentage of total and PCR-positive cases per racetrack location

Of these samples, 42 (22%) were PCR-positive for EqPV-H DNA (Fig. 1 and 2 A). Cases were submitted for necropsy throughout the year, with the highest number of submissions in spring and summer (from April through September, Table 1; Fig. 2B). However no statistical association was found between season and EqPV-H PCR status (Table 1). PCR-positive liver samples were detected in horses from seven of the ten racetracks represented with 3–74 necropsy cases per racetrack and a range of 0–67% (median of 19%) PCR-positive liver samples at each track (Table 1; Fig. 2 C), but without any statistical association between racetrack and EqPV-H PCR status (Table 1). Likewise, no statistical association was observed between EqPV-H PCR status and breed, sex, age, or cause of death (Table 1).

**Table 1:** **Demographic characteristics and association with EqPV-H infection.** Association was assessed by logistic regression. Each racetrack only had horses of one breed, therefore, association by racetrack was assessed separately by Chi-square analysis

Population	Cases (no.)	PCR Positive (no.)	PCR Positive (%)	p value
**All cases**	**191**	**42**	**22%**	
**Breed**				
Thoroughbred	157	36	23%	0.347
Standardbred	34	6	18%	
**Sex**				
Stallion	32	5	16%	0.587
Gelding	87	20	23%	
Mare	72	17	24%	
**Age**				
Median (range)	4 (2–13)	4.5. (2–10)		0.688
**Cause of death**				
Musculoskeletal injury	119	26	22%	0.839
Other causes	72	16	22%	
**Submission period**				
Spring (April-June)	58	15	26%	0.856
Summer (July-Sept)	76	15	20%	
Fall (Oct-Dec)	27	6	22%	
Winter (Jan-Mar)	30	6	20%	
**Racetrack**				
A (TB)	74	16	22%	0.204
B (SB)	3	0	0%	
C (TB)	31	8	26%	
D (TB)	35	10	29%	
E (TB)	17	2	12%	
F (SB)	4	2	50%	
G (SB)	12	2	17%	
H (SB)	3	2	67%	
I (SB)	7	0	0%	
J (SB)	5	0	0%	

### Equine parvovirus-hepatitis (EqPV-H) infection in racehorses is associated with hepatitis

**Fig. 3:**
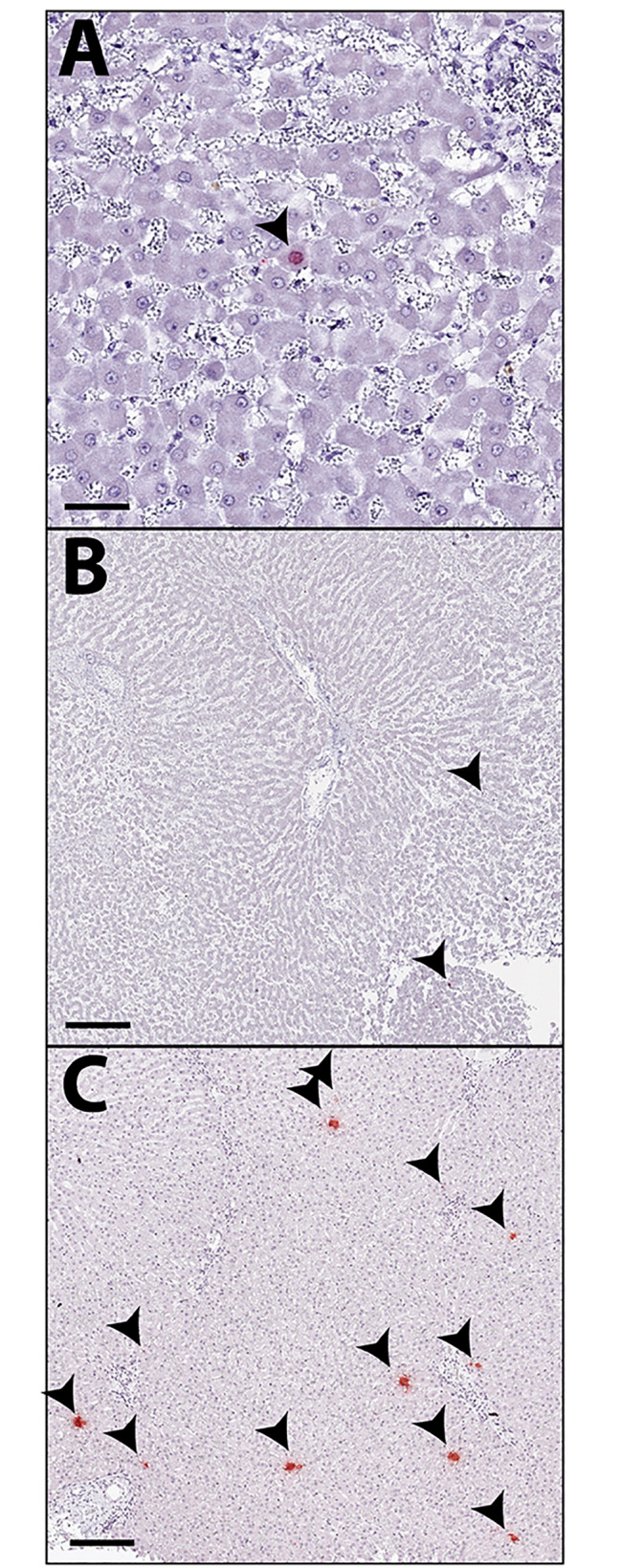
***In situ*hybridization (ISH) distribution in horse liver samples. (A).** Case 25 represents cases of positive ISH with only 1–2 positive cell (arrowhead) per section. **(B).** Case 34 represents cases of positive ISH with 3–10 positive cells per section, with two cells highlighted here (arrowheads). **(C).** Case 4 represents cases of positive ISH with multiple positive hepatocytes (arrowheads) throughout the section Scale bar A = 400 μm, B and C = 200 μm. EqPV-H ISH probe

To determine whether detection of EqPV-H DNA was associated with viral infection of hepatocytes, in situ hybridization (ISH) was performed on liver tissue sections that tested positive for EqPV-H DNA by PCR (Fig. 1). Of the 42 PCR-positive samples, 31 (73.8%) demonstrated positive hybridization in hepatocytes (Fig. 1; Table 2). The majority of hybridization was observed in lobular hepatocytes, though rare hybridization within or adjacent to portal tracts was also detected. Of the 31 ISH-positive samples, 15 (48.4%) had hybridization in only one or two cells per section, and in the cases of single cell hybridization, positive puncta were often only identified in the nucleus (Fig. 3 A); 11 (35.4%) had multiple positive cells per section (Fig. 3B), and 5 (16%) samples had more widespread positive hybridization throughout the section with at least 1–3 ISH-positive cells per hepatic lobule (Fig. 3 C).

**Table 2:** **ISH information and presence/absence of hepatitis of the 42 Equine parvovirus-hepatitis (EqPV-H) PCR-positive cases.** S, stallion; M, mare; G, gelding; NA, not assessed; MS, musculoskeletal; TB, Thoroughbred; SB, Standardbred

Case	ISH score	ISH signal in necrotic cells or associated with inflammation	Hepatitis
**1**	+++	+	yes
**2**	+++	+	yes
**3**	+++	+	yes
**4**	+++	+	yes
**5**	++	+	yes
**6**	++	+	yes
**7**	++	+	yes
**8**	++	+	yes
**9**	++	-	yes
**10**	++	-	yes
**11**	+	-	yes
**12**	+	-	yes
**13**	+	-	yes
**14**	+	-	yes
**15**	+	-	yes
**16**	+	-	yes
**17**	+	-	yes
**18**	+	-	yes
**19**	+	-	yes
**20**	-	-	yes
**21**	-	-	yes
**22**	-	-	yes
**23**	-	-	yes
**24**	++	+	no
**25**	+	-	no
**26**	+	-	no
**27**	+	-	no
**28**	-	-	no
**29**	-	-	no
**30**	+++	+	NA
**31**	++	NA	NA
**32**	++	NA	NA
**33**	++	NA	NA
**34**	++	+	NA
**35**	+	NA	NA
**36**	+	NA	NA
**37**	+	NA	NA
**38**	-	NA	NA
**39**	-	NA	NA
**40**	-	NA	NA
**41**	-	NA	NA
**42**	-	NA	NA

**Fig. 4:**
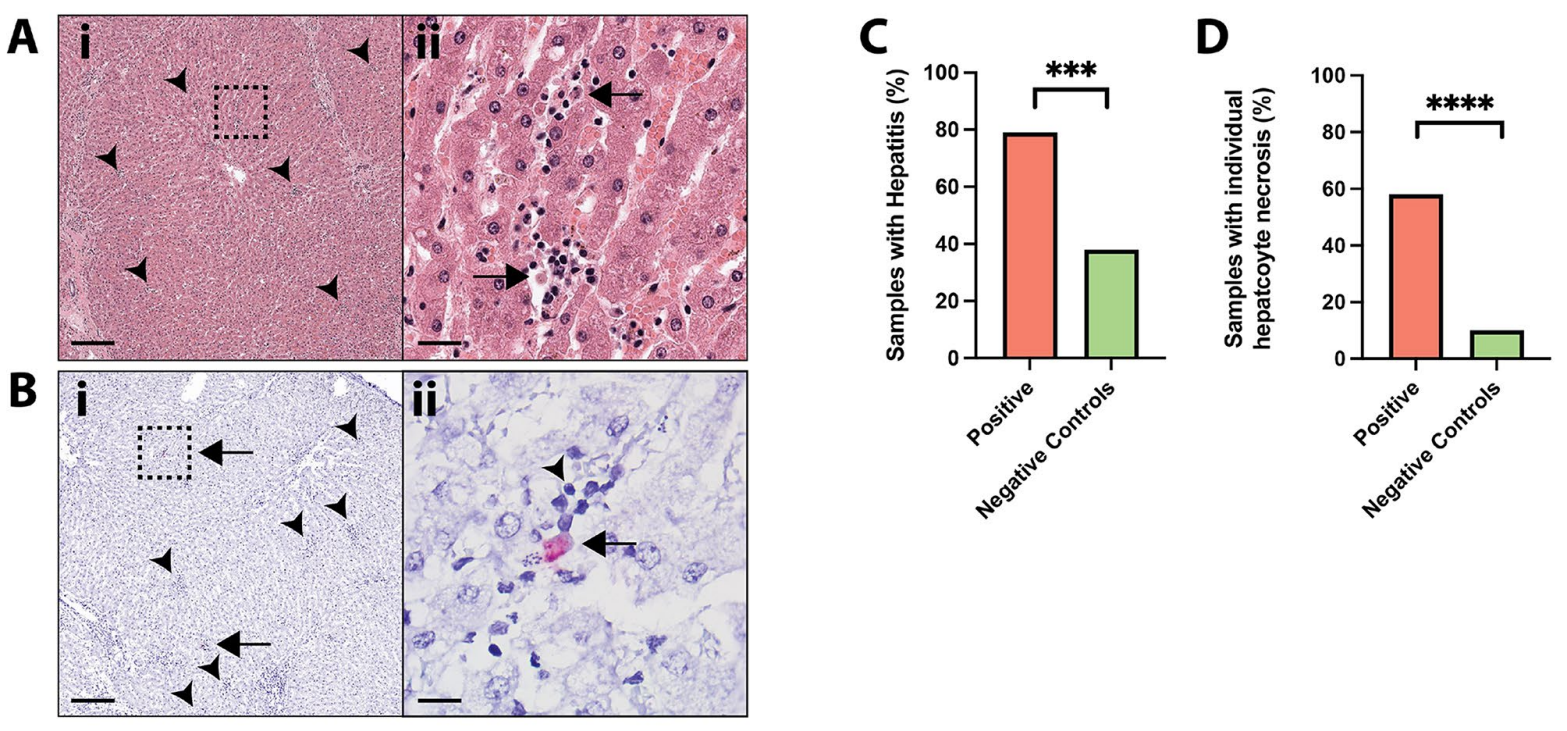
**Equine parvovirus-hepatitis (EqPV-H) infection in racehorses is associated with hepatitis. (A).** Assessment of Case 1 by H&E shows multifocal lobular infiltrates of inflammatory cells (arrowheads), consistent with hepatitis **(i)**. The highlighted region of A shows examples of shrunken necrotic cells associated with inflammatory infiltrates (arrows) **(ii)**. **(B).** Assessment of Case 2 by ISH shows positive hybridization (arrow) associated with multifocal inflammatory infiltrates (arrowhead) in hepatic lobules **(i).** The highlighted region of B shows hybridization in a necrotic cell (arrow) associated with multiple small lymphocytes (arrowhead) **(ii). (C)**. Histologic evidence of hepatitis was higher in EqPV-H PCR-positive cases compared to EqPV-H PCR-negative controls. **(D).** Histologic evidence of individual hepatocyte necrosis was higher in EqPV-H PCR-positive cases compared to EqPV-H PCR-negative controls. Scale bar A(i) and B(i) = 200 μm; A(ii) = 20 μm; B(ii) = 10 μm. ***: p = 0.0005; ****: p < 0.0001

PCR-positive liver tissues were also stained with H&E for histopathology analysis, with 29 samples of sufficient histological quality to be analyzed (Fig. 1). Of those, 23 sample sections showed signs of hepatitis (79%), defined as having more than two lobules with focal inflammatory cell infiltrates, and 17 sample sections had individual hepatocyte necrosis (59%). Inflammatory cell infiltrates were composed predominantly of small lymphocytes, with satellitosis around shrunken, necrotic cells (Fig. 4 A). Combining the histology of these 29 samples with ISH showed that 11 sample sections had hybridization in necrotic cells that were associated with inflammatory cell infiltrates (48%) (Fig. 4B).

Interestingly, when comparing liver samples with signs of hepatitis between PCR-positive and PCR-negative samples, a significantly higher percentage of hepatitis was seen in PCR-positive liver samples (23 out of 29; 79%) versus PCR-negative samples (20 out of 52; 38%) (Fig. 4 C; p = 0.0005). Moreover, there was a significantly higher proportion of samples with individual hepatocyte necrosis in PCR-positive, ISH-positive liver samples (17 out of 29; 59%) versus PCR-negative samples (5 out of 52; 10%) (Fig. 4D, p < 0.0001). Of the 5 cases with widespread positive hybridization throughout the section with at least 1–3 ISH-positive cells per hepatic lobule (Fig. 1 and 3 C), all but one had lobular hepatitis.

### Equine parvovirus-hepatitis (EqPV-H) was detected in horses with and without musculoskeletal injuries

As part of the NYSGC program examining racetrack injuries, all horses that die or are euthanized on racetracks are submitted for a complete postmortem examination. Of the horses included in this study, 119 cases were submitted due to musculoskeletal injuries sustained during performance, whether racing or training (Table 1). However, no statistical association was found between EqPV-H PCR status and death from musculoskeletal injury versus those from other causes of death (p = 0.839; Table 1). Illnesses reported in horses with ISH-positive liver samples that were not related to musculoskeletal injury included colic, pleuritis, sudden death, and encephalitis (Suppl Table 1).

## Discussion

The goal of this study was to determine the prevalence and pathology associated with EqPV-H infection in racehorses from New York racetracks This study demonstrates that EqPV-H is both prevalent and pathogenic in this population, based on the presence of viral nucleic acid within necrotic hepatocytes associated with inflammatory cell infiltrates. We found an overall prevalence of 22% in liver samples of racehorses from New York racetracks, which is similar, albeit lower, to the 37% prevalence that was previously observed in serum samples from Thoroughbreds in New York, Florida, and Kentucky [10] and the 15% serum prevalence previously found in equine serum samples submitted to the AHDC at Cornell University [1]. Considering the potential for DNA degradation during FFPE processing and storage, the true prevalence in our study population might actually be higher, and thus, more closely resembling the study of Mann et al., 2021 [10]. Interestingly, studies in South Korea and China examining EqPV-H infection in apparently healthy racehorses found much lower prevalence rates of 4.2% and 8.33%, respectively, based on serum PCR [6, 14], indicating that EqPV-H prevalence could vary by region although more data are needed to address this.

EqPV-H infection is reported to vary with sex and age. The study in South Korea found an association of EqPV-H infection with sex, with stallions and geldings being more affected compared to mares [14]. The study in China found that out of the 60 horses sampled, all 5 EqPV-H-positive horses were stallions or geldings, although no formal statistical analysis of sex association was performed [6]. Related to age, a study of 259 horses in Austria identified a significantly higher probability of EqPV-H DNA detection in 16- to 31-year-old horses when compared to both 1- to 8-year-old and 9- to 15-year-old horses [9]. In our study, no statistically significant association was found between EqPV-H infection and either sex or age. For the latter, it has to be noted that our population consisted of racehorses in the 2- to 13-year-old age range (median age of 4), so no comparisons could be made with older horses as those were not represented in our study.

Since our study only included racehorses from NYS racetracks, an analysis of epidemiologic factors that could potentially contribute to infection was not performed. Nonetheless, the relatively consistent distribution of EqPV-H-positive cases across the different racetracks further supports the endemic nature of this virus, and was corroborated by a lack of a statistically significant association between EqPV-H infection status and racetrack. The 3 racetrack facilities that did not have any PCR-positive cases only had very few cases submitted during the study period (between 3 and 7 cases, with a median of 5). Given the relatively high overall prevalence of EqPV-H in horse populations reported so far, combined with the frequent movement of performance horses to different facilities, makes it likely that EqPV-H is present at most, if not all locations with large numbers of horses.

Aside from the demonstration of viral nucleic acid in the liver of Theiler’s disease cases and experimentally infected horses, the role of EqPV-H in naturally occurring hepatopathies remains elusive. A recent study of DNA extracted from FFPE liver tissues of 84 liver disease cases in horses and donkeys in the Austria identified two EqPV-H PCR-positive liver samples [19], both involving neoplastic liver metastases. However, the low rate of EqPV-H detection could reflect low infection prevalence, as the prevalence of EqPV-H in horses in the Austria has not been formally reported. Here, we describe histopathologic evidence of mild hepatitis associated with naturally occurring EqPV-H infection in racehorses. The main histologic features of infected cases were mild lymphocytic lobular hepatitis and individual cell necrosis. Importantly, these findings were highly similar to the mild hepatitis described in experimental EqPV-H infections, suggesting that these experimental models accurately recapitulate natural disease [1, 2].

Using ISH, only 31 of the 42 PCR-positive cases were positive for viral NA (74%). Although somewhat unexpected, this could be explained by false positive PCR results and/or differences in sample source. For example, a single infected cell found in some of the cases, as further discussed below, may be captured by three 10 μm-thick scrolls of FFPE liver tissue used for DNA extraction and PCR, but missed in a single 4 μm section slide of liver tissue used for ISH. Interestingly, we found only a single hepatocyte out of tens of thousands to be positive for viral nucleic acid by ISH in some of our EqPV-H PCR-positive liver samples, and moreover, hybridization puncta in these single infected cells were often small and single, suggesting a low copy number of virus DNA [20]. Whether these single infected hepatocytes represent an early, subclinical phase of the infection or a more chronic persistent form, is unclear. Since individual hepatocytes with positive hybridization for EqPV-H viral nucleic acid have been described in persistently infected horses (defined as detectable viral loads in livers > 15 weeks after infection) after EqPV-H experimental infections [2], the ISH pattern of individual positive cells observed in the liver of the necropsied racehorses in our present study might be indicative of chronic persistent EqPV-H infections.

## Conclusion

This study demonstrates that EqPV-H is prevalent in racehorses from New York racetracks and is associated with mild liver pathology. Moreover, it corroborates findings in EqPV-H-experimentally infected horses, where infection most commonly results in mild hepatitis. Additional studies on potential associations between EqPV-H infection and racing performance are warranted to further understand the impact of EqPV-H on the racehorse industry.

## Electronic supplementary material

Below is the link to the electronic supplementary material.

Supplementary Material 1

## Data Availability

The datasets used and/or analyzed during the current study are available from the corresponding author on reasonable request.

## List of abbreviations

EqPV-H: Equine Parvovirus-hepatitis.
GGT: Gamma-glutamyl transferase.
NYRA: New York Racing Association.
AHDC: Animal Health Diagnostic Center.
NYSGC: New York State Gaming Commission.
FFPE: Formalin-fixed paraffin-embedded.
ISH: In situ hybridization.
H&E: Hematoxylin and eosin.
MS: Musculoskeletal.
TB: Thoroughbred.
SB: Standardbred.
NA: Not assessed.
