# Supplementary material for: Prevalence and pathology of equine parvovirus-hepatitis in racehorses from New York racetracks
Source: Virol J. 2022 Nov 1;19:175. doi: 10.1186/s12985-022-01901-3 (PMC9628030; doi:10.1186/s12985-022-01901-3)
Supplement: Supplementary file 1 — Supplementary Material 1 [file 12985_2022_1901_MOESM1_ESM.docx]

**Supplemental Table 1. Demographic information, manner of death, and racetrack information of the 42 Equine parvovirus-hepatitis *(*EqPV-H) PCR-positive cases.** S, stallion; M, mare; G, gelding; TB, Thoroughbred; SB, Standardbred; E, euthanized; SD, sudden death; MS, musculoskeletal.

| **Case** | **Age** | **Sex** | **Breed** | **Manner of death** | **Racetrack** | **MS injury** | **Other illnesses** |
| --- | --- | --- | --- | --- | --- | --- | --- |
| **1** | 6 | G | TB | E | A | yes | - |
| **2** | 4 | F | TB | E | C | yes | - |
| **3** | 8 | G | TB | E | A | no | pleuritis |
| **4** | 3 | F | TB | E | A | no | encephalitis |
| **5** | 4 | M | TB | E | A | yes | - |
| **6** | 7 | M | TB | E | A | yes | - |
| **7** | 6 | F | TB | E | A | yes | - |
| **8** | 2 | G | SB | death | G | yes | - |
| **9** | 3 | M | TB | E | D | yes | - |
| **10** | 6 | G | SB | E | G | no | meningitis |
| **11** | 3 | G | TB | E | D | yes | - |
| **12** | 3 | M | TB | E | A | yes | - |
| **13** | 8 | G | SB | SD | H | no | colic |
| **14** | 3 | M | TB | SD | A | no | colitis |
| **15** | 3 | M | TB | E | A | yes | - |
| **16** | 3 | G | TB | E | D | yes | - |
| **17** | 2 | F | TB | E | E | yes | - |
| **18** | 6 | G | TB | E | A | yes | - |
| **19** | 2 | M | TB | SD | C | no | SD |
| **20** | 4 | G | TB | E | D | Yes | - |
| **21** | 5 | F | TB | E | C | Yes | - |
| **22** | 3 | F | TB | E | D | Yes | - |
| **23** | 2 | F | TB | E | C | Yes | - |
| **24** | 6 | G | TB | E | E | no | colic |
| **25** | 4 | S | TB | SD | A | no | colic |
| **26** | 9 | G | TB | SD | C | no | colic |
| **27** | 6 | G | TB | E | C | yes | - |
| **28** | 5 | F | TB | E | D | Yes | - |
| **29** | 5 | G | TB | death | A | No | enteritis |
| **30** | 7 | F | TB | SD | C | yes | - |
| **31** | 6 | G | TB | E | F | yes | - |
| **32** | 3 | G | TB | E | A | yes | - |
| **33** | 7 | F | SB | death | F | no | unknown |
| **34** | 10 | G | TB | E | D | yes | - |
| **35** | 8 | F | TB | E | D | yes | - |
| **36** | 2 | F | TB | E | A | yes | - |
| **37** | 2 | M | TB | E | A | yes | - |
| **38** | 6 | G | TB | death | A | No | colic |
| **39** | 6 | F | SB | E | H | Yes | - |
| **40** | 3 | G | TB | E | D | Yes | - |
| **41** | 4 | G | TB | E | D | Yes | - |
| **42** | 5 | G | TB | SD | C | Yes | - |
